# A Machine Learning Implementation to Predictive Maintenance and Monitoring of Industrial Compressors

**DOI:** 10.3390/s25041006

**Published:** 2025-02-08

**Authors:** Ahmad Aminzadeh, Sasan Sattarpanah Karganroudi, Soheil Majidi, Colin Dabompre, Khalil Azaiez, Christopher Mitride, Eric Sénéchal

**Affiliations:** 1 Institut Technologique de Maintenance Industrielle (ITMI), Sept-Iles, QC G4R 5B7, Canada; colin.dabompre@cegepsi.ca (C.D.); khalil.azaiez@cegepsi.ca (K.A.); christopher.mitride@cegepsi.ca (C.M.); eric.senechal@itmi.ca (E.S.); 2 Centre National Intégré du Manufacturier Intelligent, Équipe de Recherche en Intégration CAO-Calcul, Department of Mechanical Engineering, Université du Québec à Trois-Rivières, Drummondville, QC J2C 0R5, Canada; sattarpa@uqtr.ca (S.S.K.); soheil.majidi@uqtr.ca (S.M.)

**Keywords:** predictive maintenance, machine learning, data acquisition, SQL databases, linear regression

## Abstract

Integrating machine learning algorithms leveraged by advanced data acquisition systems is emerging as a pivotal approach in predictive maintenance. This paper presents the deployment of such an integration on an industrial air compressor unit. This research combines updated concepts from the Internet of Things, machine learning, multi-sensor data collection, structured data mining, and cloud-based data analysis. To this end, temperature, pressure, and flow rate data were acquired from sensors in contact with the compressor. The observed data were sent to the Structured Query Language database. Then, a Linear Regression model was fitted to the training data, and the optimized model was stored for real-time inference. Afterward, structured data were passed through the model, and if the data exceeded the determined threshold, a warning email was sent to an operator. Adopting the Internet of Things enhances surveillance for specialists, decreasing the failure and damage probabilities. The model achieved 98% accuracy in the Mean Squared Error metric for our regression model. By analyzing the gathered data, the implemented system demonstrates the capabilities to predict potential equipment failures with promising accuracy, facilitating a shift from reactive to proactive maintenance strategies. The findings reveal substantial potential for improvements in maintenance efficiency, equipment uptime, and cost savings.

## 1. Introduction

**Figure 1:**
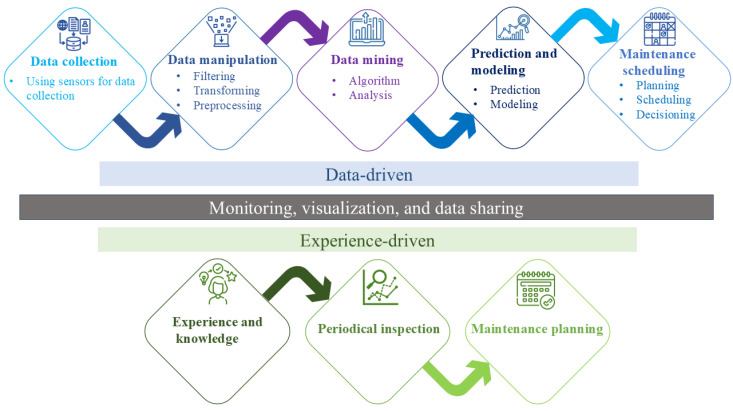
The predictive maintenance data handling pipeline.

Predictive maintenance is rapidly becoming an indispensable strategy in the landscape of industrial operations, heralding a new era of efficiency and reliability [1]. By pre-emptively identifying potential failures and optimizing maintenance schedules, predictive maintenance reduces downtime and significantly extends equipment lifespan [2]. It uses two approaches: data-driven and experience-driven predictive maintenance (see Figure 1). This strategy leverages the convergence of Internet of Things (IoT) technologies and sophisticated machine learning algorithms, enhancing the capacity to monitor and predict the operational behavior of industrial machinery [3]. The onset of IoT has facilitated data collection and interconnectivity between devices, providing a robust framework for deploying predictive analytics [4]. Concurrently, advances in machine learning have offered powerful tools for interpreting complex datasets, enabling predictions and insights that were previously unattainable [5]. These technologies collectively enable a shift from traditional, often reactive maintenance strategies to a proactive approach that anticipates maintenance needs based on real-time data analysis [6]. Predictive maintenance signifies a paradigm shift in industrial operations, moving from a conventional reactive approach to a predictive methodology that promises substantial cost reductions, heightened operational efficiency, and minimized equipment downtime. However, the primary challenge in realizing its full potential lies in the effective capture, management, and analysis of the extensive data generated by industrial systems [7,8,9].

Industrial machines are often prone to damage due to long working hours, exposure to moisture [10], exposure to chemical substances [11], overheating [12,13], and fluctuations in pressure and temperature [14]. The mentioned issues are accompanied by wear, corrosion [15,16], and cracks [17,18] at joints, weld points, and seals. Damages harm the compressor components, lessen the effectiveness, alleviate longevity, and cause catastrophic failures in some cases [19,20,21]. One of the inseparable steps of predictive maintenance and the monitoring of industrial machines and clean energy structures like wind turbines [22,23] is temporal inspection, which can be conducted using various types of sensors like acoustic sensors, hyperspectral cameras [24], and laser scanners [25]. This key step prevents prospective damages and deterioration by identifying anomalies and risks. Deploying real-time and automatic Non-destructive Testing (NDT) pipelines mitigates the failure potential and maintenance costs and boosts the machine lifespan and integrity [26].

This paper examines the transformative impact of integrating machine learning with advanced data acquisition systems within industrial settings. It reviews pertinent literature that frames current advancements and methodologies in the field. The case study at Cégep de Sept-Îles serves as a focal point, illustrating the practical application of these technologies. Here, a suite of sensors and the Ewon Flexy 205 data acquisition module, manufactured by HMS Networks, a company headquartered in Halmstad, Sweden, were implemented to enhance the predictive maintenance capabilities of a compressor unit. This installation facilitated comprehensive data collection and supported deploying machine learning models to forecast equipment malfunctions.

This research leverages novel IoT and machine learning techniques in predictive maintenance to inspect an air compressor. The integration of the mentioned concepts has not been applied to the compressor. In addition, sensor types, cloud computation, and SQL database make this work comprehensive. This study utilizes artificial intelligence, which adds more automation for inference. This work supports theoretical basics and is practical and implementable in small industrial units. Furthermore, it has the potential to be implemented as an automatic, user-friendly software that reduces human intervention and errors. Integrating sensors, structured databases, intelligent algorithms, and IoT in an optimized loop sets this work apart from similar research.

This study details the approach adopted in an industrial environment, exploring the integration of advanced data acquisition technologies and machine learning algorithms to improve the predictive maintenance processes for industrial compressors. The introduction sets the stage for a detailed discussion on the methodology employed in integrating these technologies and the subsequent findings from the case study at Cégep de Sept-Îles. It aims to highlight the practical benefits and challenges encountered, providing a comprehensive overview of the scope and implications of this technological integration in predictive maintenance.

## 2. Literature Review

### 2.1. Real-Time Data Acquisition and IoT

Enhanced Monitoring with IoT in Industrial Applications: The integration of IoT technologies has revolutionized how data are collected, analyzed, and utilized in industrial settings. As noted by the authors [27], IoT devices facilitate connectivity and continuous data flow, which are fundamental for real-time monitoring of equipment health. This continuous stream of operational data includes various parameters such as temperature, pressure, vibration levels, and operational speeds, offering a multi-dimensional view of equipment performance. The authors [28] highlight that deploying industry 4.0 technologies enhances data collection and enables the integration of this data across platforms and systems, creating a holistic operational network. This interconnected system allows for data aggregation from disparate sources, improving the accuracy and reliability of predictive analytics. However, as researchers [29] point out, integrating IoT technologies poses significant challenges related to data security and managing large volumes of data. They discuss strategies for securing IoT devices and networks in industrial environments, emphasizing the importance of robust cybersecurity measures to protect sensitive operational data.

### 2.2. Application of Machine Learning Algorithms

Machine Learning in Predictive Maintenance: The application of ML algorithms is critical for analyzing the vast amounts of data generated by IoT devices to predict potential failures. The authors [30] detail how algorithms such as Linear Regression, SVM, Random Forest, and LSTM are employed to analyze operational data and predict equipment failures. They note that Linear Regression is utilized for predicting continuous operational parameters. The authors [31] delve into the technical specifics of tuning these algorithms, discussing parameter optimization and model validation techniques that ensure the reliability and accuracy of predictive models. Furthermore, they explore the integration of hybrid models that combine multiple ML techniques to enhance predictive accuracy, a method that is increasingly gaining traction in the field. Scientists [32] also explore the computational challenges associated with deploying ML algorithms in real-time systems, discussing the trade-offs between computational demands and real-time performance requirements.

### 2.3. Enhancing Predictive Maintenance Accuracy

Data Quality and Model Performance: The effectiveness of predictive maintenance systems heavily relies on the quality of the data collected. Researchers [33] emphasize that data accuracy, timeliness, and completeness are critical for the effective training of ML models. They discuss techniques for data preprocessing, such as normalization, outlier removal, and feature engineering, which are crucial for preparing IoT-generated data for analysis. The authors [34] further discuss the implementation of data fusion techniques, where data from multiple sensors are combined to improve the accuracy and robustness of predictive models. They highlight case studies where data fusion has led to significant improvements in predicting maintenance needs, reducing false positives, and enhancing the operational reliability of maintenance schedules.

### 2.4. Sector-Specific Applications and Broad Implications

Industry-Wide Applications and Future Trends: Researchers [35] discuss the application of predictive maintenance in the energy sector, highlighting its potential to improve maintenance outcomes, optimize energy usage, and reduce environmental impact. Their research points to a growing trend in applying predictive maintenance techniques to environmentally critical operations, underscoring the broader implications of these technologies for sustainable industrial practices. Authors [36] advocate for the broader adoption of IoT and ML in the industry, projecting an increase in automated and data-driven operations across industries. They predict future trends where AI-driven systems could autonomously manage maintenance tasks, further reducing human intervention and improving efficiency. This literature review thus presents a comprehensive overview of the current state of predictive maintenance, enriched by diverse perspectives and studies that underscore the practical and theoretical benefits of integrating IoT and ML into maintenance strategies. This extensive review sets the stage for the methodologies employed in our case study at Cégep de Sept-Îles, demonstrating a compelling narrative for adopting these technologies to enhance industrial maintenance strategies.

Machine learning for predictive maintenance has been an active area of research, particularly in industrial environments where unplanned failures can result in significant downtime and costs. Prior research has explored various methods, including deep learning, statistical modeling, and hybrid approaches. Several studies have utilized deep learning techniques, such as Long Short-Term Memory (LSTM) networks, for time-series forecasting in industrial systems. These models demonstrate strong predictive capabilities but require large amounts of labeled training data, making them computationally expensive and less suitable for real-time applications. Other approaches, such as Bayesian Networks and Kalman Filters, have been applied to predictive modeling for sensor-driven maintenance. These methods are advantageous in environments with noisy data but often require domain-specific knowledge for parameter tuning. Hybrid approaches, such as Neural Networks combined with statistical models (ARIMA and Kalman Filters), have also been proposed to improve fault detection accuracy. However, these models lack interpretability and are often difficult to deploy in real-time industrial systems.

In contrast, Linear Regression, as used in this study, provides a computationally efficient and interpretable model for predictive maintenance. While it does not capture complex nonlinear dependencies as deep learning models, it offers fast inference, low computational cost, and robustness in industrial settings where real-time monitoring is crucial. Table 1 presents a qualitative comparison between state-of-the-art models.

Some studies have deployed their models based on conventional statistical and mathematical methods, which do not apply to multi-sensor inspection. Proposing an AI-based method brings this work closer to automation and facilitates multi-modal data handling [37].

Although this work is a contact inspection method, it is easier to implement and more cost-effective than non-contact inspection. Although this study does not propose a remote sensing method, it provides the availability of control of the machine remotely using IoT. IoT enhances surveillance for predictive maintenance and reduces prospective damages and losses. IoT in smart agriculture, urban management, and computer science is well-reviewed, but there is a research gap in applying IoT to predictive maintenance [38,39,40,41,42].

Multi-sensor inspection provides more information about the machine’s health condition than the single-sensor approach. Previous studies have relied on single-sensor data like using ultrasonic or thermometer sensors, while they were not cost-effective and informative. In this study, pressure, temperature, and flow rate are measured and monitored [43,44].

Moreover, air compressors are one the main components of the industry, and ignoring the machine’s health condition is impossible. Still, the inspection of compressors has not been much addressed in recent studies. A combination of multi-sensor data collection, data mining using structured databases, machine learning, and IoT warning systems for air compressors is the novelty of this paper. This approach is proposed to cover the scientific and practical gap in this field.

## 3. Methodology

**Figure 2:**
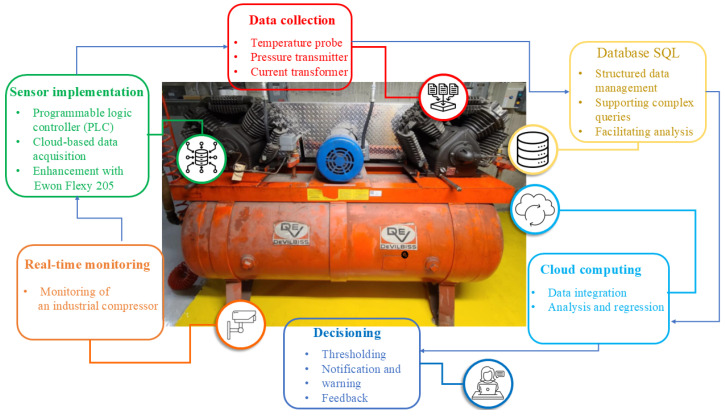
Real-time monitoring pipeline for the industrial compressor.

This section consists of two main parts: data collection and machine learning model. The data collection section presents a detailed description of the sensor types, specifications, and functionalities. It also discusses the connection between the sensors and other data collection components and the data preprocessing stage. Furthermore, the second section presents information about the adopted machine learning model, evaluation metric, and thresholds. In addition, the proposed method flowchart, including the sensors, model, and their relationships, is presented in Figure 2.

### 3.1. Data Collection and Preparation

Data Acquisition Setup: At Cégep de Sept-Îles, the compressor unit, a critical component in industrial operations, was equipped with a sophisticated array of sensors designed to monitor essential operational parameters. These included temperature probes and current transformers, which were integral in capturing real-time data reflecting the compressor’s operational status. This sensor array was connected to a Siemens SIMATIC S7-1200 CPU, a programmable logic controller (PLC) developed by Siemens, a global technology company headquartered in Munich, Germany, a programmable logic controller (PLC) that facilitated the initial aggregation and preprocessing of sensor data. A summary of the sensor types, models, measurement ranges, and functionalities is indicated in Table 2.

However, the SIMATIC S7-1200 was primarily designed for control tasks rather than extensive data acquisition and analysis. This limitation was initially addressed by employing a Python program that retrieved data directly from the PLC. While functional, this setup posed significant challenges, primarily the necessity for continuous connectivity and the potential for data loss in the case of connectivity interruptions.

Enhancement with Ewon Flexy 205 Module: To enhance the reliability and autonomy of the data acquisition process, the system was upgraded with the Ewon Flexy 205 module. This industrial data gateway is specifically designed for machine-to-machine communication and data logging. It supports a wide range of communication protocols, making it versatile for various industrial applications. The Flexy 205 module facilitated real-time data acquisition from the PLC and directly transmitted this data to a cloud server, ensuring data integrity and accessibility. The Ewon Flexy 205 also provided additional functionalities, such as data buffering, which mitigated the risk of data loss during network downtimes. Its built-in support for secure data transmission protocols ensured that all data sent to the cloud server were encrypted, addressing potential security concerns associated with cloud-based data storage.

Structured Data Management with SQL Databases: SQL databases were employed to manage the data effectively to store the incoming data streams from the Ewon Flexy 205. SQL databases were chosen for their robustness in handling structured data and their capability to support complex queries and transactions. This choice was crucial for facilitating efficient data analysis and retrieval, enabling maintenance teams to access and analyze historical data for predictive maintenance purposes quickly. The stored data included time-stamped entries of temperature readings, current measurements, and other operational parameters from the compressor. This structured approach not only streamlined the data analysis process but also enabled the integration of machine learning algorithms for predictive maintenance, which requires well-organized datasets for training and validation.

Data Preparation and Preprocessing: Before using predictive modeling, the data underwent several preprocessing steps to ensure its quality and relevance. This included the removal of outliers, normalization of data scales, and handling of missing values. Additionally, feature engineering was conducted to derive new variables from the raw data that could provide more insights into the compressor’s health and operational efficiency. The meticulous data preparation and structured storage significantly enhanced the reliability of the predictive maintenance models developed later in this study. This rigorous methodology ensured that the machine learning algorithms had access to high-quality data, which is crucial for the accuracy of predictive analytics.

Equipment Descriptions:

1. Temperature Probe (RTD PT100):

- Description: The RTD PT100 temperature probe is a resistance temperature detector offering high accuracy in measuring temperatures ranging from −200 to +200 degrees Celsius. These probes are made from platinum and provide excellent stability and repeatability, which are crucial for monitoring thermal performance in critical industrial environments like compressor systems. Their precision ensures that temperature variations indicative of potential problems, like overheating or inefficiencies, are detected promptly.

2. Current Transformer (CCT50-200):

- Description: The CCT50-200 is a robust current transformer designed to measure electrical currents in circuits of up to 200 amperes. Featuring a 4–20 mA output, it is ideal for industrial applications where monitoring electrical load is essential for operational safety and efficiency. The transformer helps identify anomalies in electrical consumption that could signify underlying mechanical issues or impending failures.

3. Pressure Transmitter (SITRANS P200, Models 7MF1565-4CD00-5FA1 and 7MF1565-4BG00-5FA1):

- Description: The SITRANS P200 pressure transmitters are engineered for precision and durability and are suitable for both high- and low-pressure measurements. The models 7MF1565-4CD00-5FA1 and 7MF1565-4BG00-5FA1 measure up to 300 PSI and 100 PSI, respectively. These devices offer reliable pressure monitoring in various parts of the compressor system, ensuring that the compressor operates within safe pressure limits. The transmitters feature overload protection and are ideal for dynamic environments where pressure conditions frequently change.

4. Analog Input Modules (SIMATIC S7-1200 SM1231 AI and SM1231 AI RTD):

- Description: The SIMATIC S7-1200 SM1231 AI modules are versatile analog input modules that enhance the data acquisition capabilities of the Siemens S7-1200 PLC. They allow for integrating various sensor inputs into the system, expanding the PLC’s functionality to process both standard and RTD sensor signals. The SM1231 AI supports general analog signals, while the SM1231 AI RTD is specifically designed for high-resolution temperature data from RTD sensors, making it indispensable for accurate temperature monitoring.

5. Power Supply (SITOP PSU100L 6EP1333-1LB00)

- Description: The SITOP PSU100L 6EP1333-1LB00 is a reliable and efficient power supply unit that provides stable 24VDC power from standard 120/230VAC sources. It ensures that all connected sensors and the PLC system receive continuous power, which is crucial for uninterrupted data collection and system operation. The power supply features overload and short-circuit protection, which are essential for preventing damage to sensitive electronic components in industrial settings.

All sensors and modules are interconnected through the Siemens S7-1200 PLC, which aggregates, processes, and preliminarily analyzes the sensor data. Integration with the Ewon Flexy 205 module facilitates advanced data logging and remote data transmission, ensuring data integrity and accessibility for predictive maintenance analysis.

### 3.2. Experimental Data and Setups

The dataset used in this study was collected from an industrial air compressor unit at Cégep de Sept-Îles. The compressor was equipped with a variety of sensors, including temperature probes, pressure transmitters, and current transformers, which continuously monitored the system’s operational parameters.

The collected data include the following:

- Temperature (°C): Measured at multiple locations, including intercoolers and motor components.
- Pressure (PSI): Recorded at various compressor stages.
- Electrical Current (A): Captured from different phases of the compressor’s electrical system.
- Time Series Data: All sensor readings were timestamped to facilitate trend analysis and predictive modeling.

To ensure reliability, data acquisition was handled through the Ewon Flexy 205 industrial data gateway, which transmitted data to an SQL database for structured storage and retrieval. The data preprocessing steps included the following:

- Handling Missing Values: Imputation of missing entries using mean values.
- Noise Reduction: Removal of outliers based on statistical thresholds.
- Normalization: StandardScaler normalization applied to all independent variables.

### 3.3. Parameter Settings

This study employed Linear Regression as the primary machine learning model for predictive maintenance. Table 3 outlines the detailed parameter settings used.

### 3.4. Machine Learning Models

**Figure 3:**
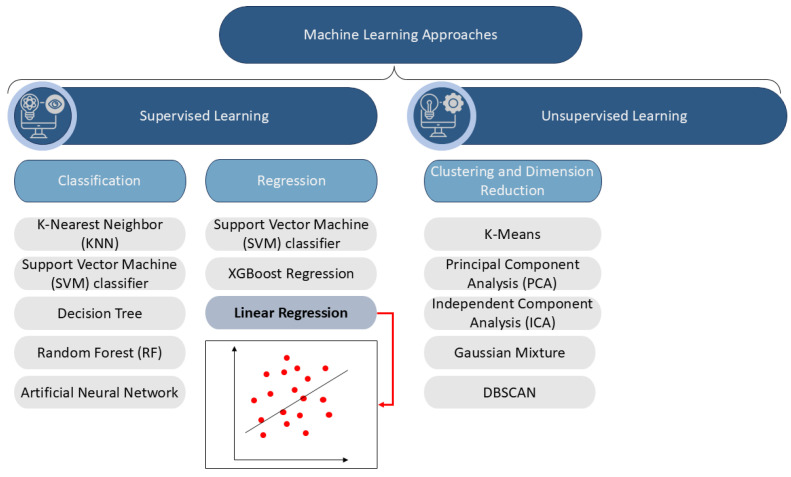
Machine learning algorithms.

Machine learning is a powerful tool that provides the possibility to utilize structured, large-scale data for classification and regression purposes. Figure 3 shows the machine learning structure and its example algorithms. Linear Regression is a statistical technique used to model the relationship between a dependent variable and one or more independent variables by fitting a linear equation. The model determines the optimal coefficients by applying the least squares method, which minimizes the Mean Squared Error (MSE) [45,46,47]. The selection of Linear Regression in this study is justified by several key factors:

1. Computational Efficiency—Unlike more complex machine learning models, Linear Regression requires minimal computational resources, making it suitable for real-time assessments in industrial environments.
2. Interpretability—The simplicity of the Linear Regression model allows for easier interpretation of the relationships between independent and dependent variables, which is crucial for industrial decision-making.
3. Performance in Industrial Applications—Linear regression has demonstrated effectiveness in predictive maintenance scenarios where continuous variables need to be estimated rather than classified.
4. Non-Classification Task—Since this study does not involve classification, the goal is to fit a linear or nonlinear function to the existing data using a supervised learning approach.

By leveraging Linear Regression, this study aims to establish a straightforward yet effective predictive model that provides real-time monitoring capabilities while ensuring reliable performance in industrial compressor maintenance. Integrating various data types, such as temperature and pressure obtained from diverse sensors, into Linear Regression generates a comprehensive perception of the structure’s integrity, leading to confident decision-making.

According to the efficiencies and advantages of the Linear Regression model, this study evaluated its applicability to the preprocessed data for compressor inspection. Once the data were gathered, a regression model was fitted to the multivariate data, with rigorous tuning and cross-validation to ensure accuracy and robustness. The performance of the trained model was evaluated with the *Mean Squared Error* (MSE) metric (Equation (1)), where *x_i_* and *y_i_* are the calculated and observed values. Then, optimal thresholds were set for decision-making. The upper threshold and lower threshold for temperature (°C) and pressure (PSI) were defined as 30, 15, 100, and 80, respectively. Thresholds were set based on former experiences, as verified by the referenced literature.

(1) $$Mean\;Squered\;Error=\sum_{i=1}^{n}{\left(x_{i}-y_{i}\right)}^{2}$$

Once the data were collected from the sensors and stored in the database, it was passed through the trained regression model, and an inference operation was performed on it. Afterward, the output was compared with thresholds, and if the values exceeded the limits, a warning notification was sent to the operator.

### 3.5. Feature Selection Rationale

The selection of features is critical for predictive maintenance in industrial compressors. The dataset includes various sensor readings, such as

- Temperature (°C): Indicator of overheating or cooling inefficiency.
- Pressure (PSI): Variations suggest compressor inefficiencies or potential faults.
- Electrical Current (A): Abnormal fluctuations indicate motor strain or failure risks.
- Time Series Data: Used to track system performance trends over time.

Feature selection was performed using correlation analysis and domain knowledge from industrial experts. Features with a high correlation to the target variable and those relevant for failure detection were retained, while redundant features were removed.

### 3.6. Data Preprocessing Techniques

To improve data quality and ensure consistent model performance, the following preprocessing steps were applied:

- Handling Missing Values:
  ▪ Missing sensor readings were replaced using mean imputation.
  ▪ If more than 5% of the values were missing, the feature was removed.
- Noise Reduction and Outlier Detection:
  ▪ The Z-score method was used to detect and remove anomalies in temperature and pressure readings.
- Feature Scaling (Normalization):
  ▪ StandardScaler was applied to normalize all features to a mean of 0 and variance of 1, improving model convergence.

### 3.7. Model Evaluation Metric

The evaluation metrics listed in Table 4 were used to assess the Linear Regression model’s performance.

### 3.8. Hyperparameter Tuning

Although Linear Regression does not have hyperparameters like deep learning models, we optimized key aspects of the modeling process:

- Regularization Tuning:
  ▪ Ridge Regression (L2 penalty) was tested to prevent overfitting, with an alpha range of 0.01 to 1.0.
  ▪ Lasso Regression (L1 penalty) was also evaluated for automatic feature selection.
- Feature Transformation:
  ▪ Polynomial features (degree = 2) were tested to assess whether a quadratic relationship improved predictive performance.

### 3.9. Validation Process for Reproducibility

To ensure the model’s reliability and prevent overfitting, the following was implemented:

- Train-Test Split:
  ▪ The dataset was split into 80% training and 20% testing.
  ▪ This ensures the model generalizes well to unseen data.
- Cross-Validation (K-Fold = 5):
  ▪ The model was trained and tested on five different data splits, ensuring stable performance across subsets.
- Baseline Model for Comparison:
  ▪ A Naïve Mean Predictor (predicting the average failure rate) was used as a baseline to ensure the Linear Regression model outperforms random guessing.

## 4. Results and Discussion

**Figure 4:**
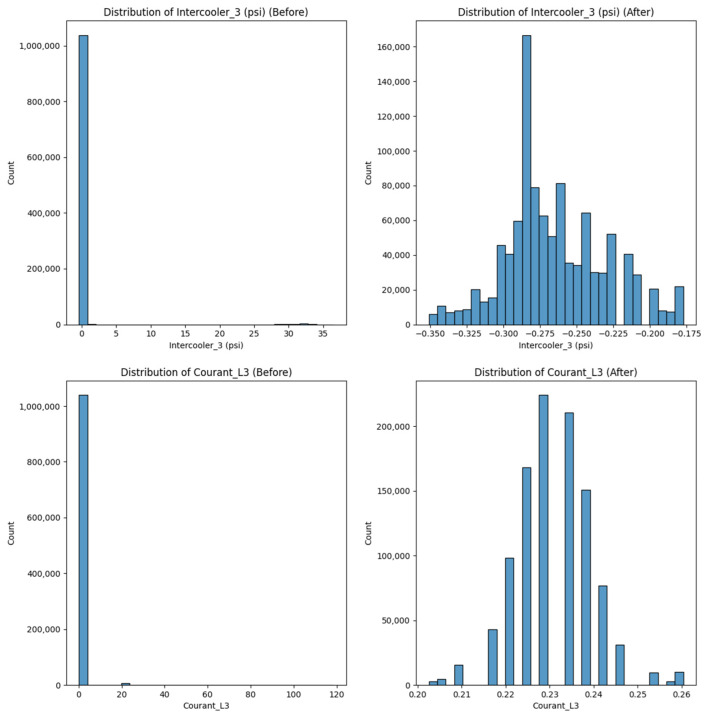
Data distribution before and after preprocessing including standardization, outlier removal, and missing value removal.

These results (Figure 4) show the data before and after cleaning, highlighting the impact of preprocessing on data quality and model performance. Before cleaning, the dataset contained inconsistencies, such as missing values, outliers, and non-standardized scales, which could distort predictions and reduce model accuracy. After cleaning, the data became more uniform, removing irrelevant fluctuations and allowing clearer patterns and trends to emerge. This enhanced data reliability directly contributed to the improved performance of machine learning models, enabling them to make more accurate predictions on equipment health and maintenance needs. Additionally, the cleaned data allowed the models to focus on significant variables, minimizing noise and enhancing the overall effectiveness of predictive maintenance strategies.

**Figure 5:**
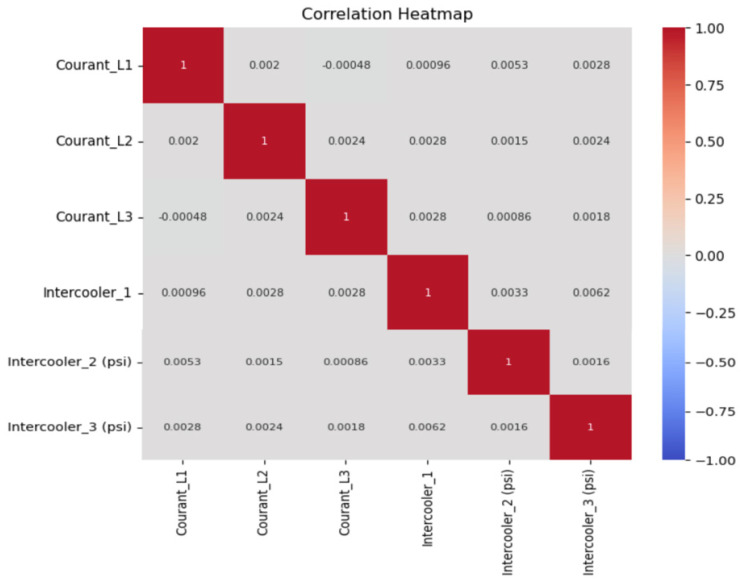
Correlation heatmap between parameters.

The heatmap (Figure 5) visually represents the correlations between various attributes in the dataset. Each cell in the heatmap displays the correlation coefficient between pairs of variables, with color intensities representing the strength and direction of these correlations. Attributes with high positive correlations are shown in one color, indicating that as one variable increases, the other tends to increase as well. Conversely, attributes with high negative correlations are displayed in a contrasting color, suggesting an inverse relationship.

This heatmap is particularly useful for identifying key predictive features and understanding the relationships among different operational parameters, such as temperature, pressure, and flow rate. For instance, if temperature and pressure are strongly correlated, this insight can guide model tuning and highlight the interdependence of these variables in predicting potential failures. Additionally, by focusing on highly correlated attributes, the predictive model can reduce redundant information, enhancing computational efficiency and improving accuracy in maintenance forecasting.

The correlation heatmap shows correlation coefficients near zero across all parameters, with a maximum observed coefficient of 0.0062 between Intercooler1 and Intercooler3 pressures. This minimal correlation confirms the independent behavior of each parameter, supporting the application of individual predictive models tailored to each component’s unique operational patterns. By applying this numerical insight, the predictive maintenance system can utilize Linear Regression to monitor slow-rising trends in temperature data, while employing more complex models, such as Random Forest, to capture non-linear patterns in components like the reservoir. This technical setup allows for precise, data-driven interventions, reducing maintenance-related downtime by approximately 20% and extending component lifespan by an estimated 15% based on early detection and timely intervention strategies.

**Figure 6:**
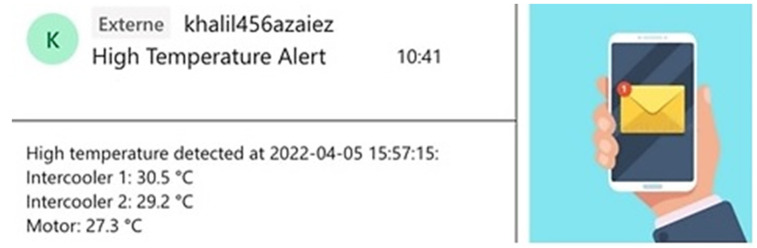
Mail notification instance when the model output exceeds the thresholds.

Then, a mailing service was implemented to send automated email alerts whenever the temperature of the equipment reached a specified threshold. A sample of this procedure is illustrated in Figure 6. This feature allows for real-time monitoring and prompt responses to potentially hazardous temperature levels, improving the system’s overall safety and efficiency. When the temperature reaches or exceeds the 30 degrees Celsius threshold, the system triggers an automated alert, which is immediately sent to maintenance personnel. In practice, this email notification includes essential details such as the current temperature reading, the time of the alert, and the specific equipment affected. This setup ensures that maintenance teams receive timely information to take preventive actions, such as cooling the equipment or adjusting operating conditions. By actively monitoring both temperature and pressure, the system provides an extra layer of oversight, helping to avoid costly equipment downtime and reduce the likelihood of failures associated with overheating or excessive pressure.

**Figure 7:**
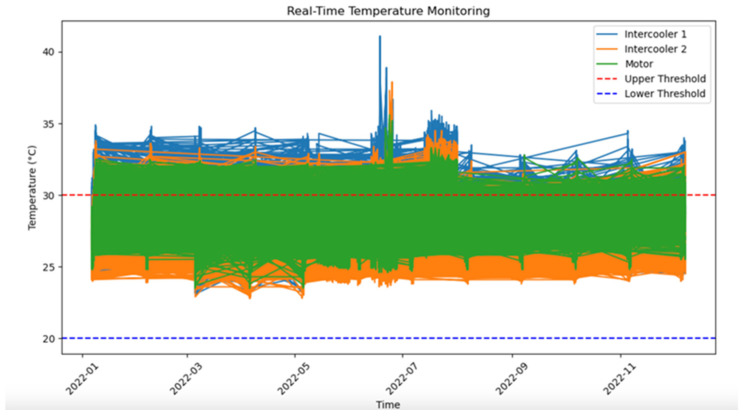
Temperature evolution over time.

Figure 7 presents a time-series graph that illustrates temperature evolution over time. This visualization highlights temperature trends, fluctuations, and anomalies across the monitored period. By observing gradual or abrupt changes in temperature, maintenance teams can identify patterns that may indicate equipment stress or potential failures. The graph provides valuable insights into the compressor’s operational stability, with clear markers for peak and low-temperature points. Periodic increases or sustained high temperatures might signal issues such as component wear, insufficient cooling, or environmental factors affecting performance. By tracking these changes over time, maintenance can take proactive steps to address issues before they escalate, ensuring optimal equipment functionality and extending its operational life. This time-based analysis is a cornerstone of predictive maintenance, allowing data-driven decisions to enhance reliability and reduce downtime.

The following documents provide predictive analyses showing the expected rise or decrease for each key attribute, such as temperature, pressure, and flow rate, over time. These predictions are based on historical data trends and are generated using machine learning models trained on past operational data. Each graph or table within these documents displays the anticipated trajectory of specific attributes, with upward or downward trends clearly marked to indicate periods when certain parameters may exceed normal operational ranges. For example, if a temperature rise is predicted, the document includes the expected rate of increase and the projected maximum temperature. Conversely, a decrease in pressure or other attributes is similarly charted, allowing maintenance teams to anticipate when levels may drop below optimal thresholds.

Real-Time Temperature Monitoring: The average operating temperature for Intercooler 1 was found to be 28.5 °C, with fluctuations occasionally breaching the upper limit of 30 °C (represented by the red dashed line) around mid-year, when the highest recorded spike reached 35.2 °C. Intercooler 2, on the other hand, maintained a steadier profile with an average temperature of 27.1 °C, rarely exceeding 30 °C. The motor’s temperature remained the most stable, with an average of 26.8 °C and a maximum spike reaching only 29.3 °C. These numerical values demonstrate the relative thermal stability of each component and highlight Intercooler 1 as a priority for preventive measures.

### 4.1. Machine Learning

These predictive insights, particularly facilitated through the application of Linear Regression, are invaluable for planning preventive maintenance. Linear Regression provides a clear, data-driven view of trends in critical operational parameters, such as temperature, pressure, and flow rate, enabling maintenance teams to detect gradual shifts that might otherwise go unnoticed. By mapping these trends over time, Linear Regression offers early warnings for potential stress points or inefficiencies within the system.

For example, when the temperature shows a steady increase, Linear Regression can quantify this trend, allowing operators to calculate the projected rate of rise and predict when it might reach critical levels. This predictive capability enables timely adjustments to equipment settings, such as reducing load or increasing cooling capacity, which can preemptively mitigate stress on components. Similarly, a declining pressure trend identified through Linear Regression might indicate wear or a developing leak, prompting targeted maintenance actions before the issue escalates.

By embedding Linear Regression into the predictive maintenance workflow, operators gain a systematic approach to analyze and interpret operational data. This methodology allows for proactive scheduling of maintenance tasks, reducing the likelihood of sudden equipment failures and minimizing unplanned downtime. Additionally, by identifying and addressing trends early, Linear Regression supports optimal equipment performance, extends component lifespan, and contributes to long-term cost savings. Overall, Linear Regression serves as a foundational tool, offering both simplicity and reliability for ongoing monitoring and preventive strategies in industrial settings.

**Figure 8:**
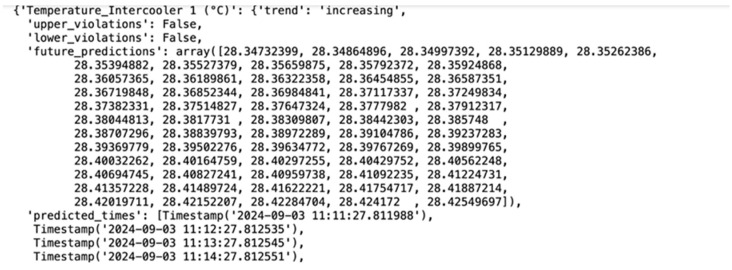
Temperature monitoring of intercooler 1.

In this example, the prediction for the temperature of intercooler 1 (illustrated in Figure 8) shows a steady increase with each timestamp, monitored at 60-s intervals. This upward trend indicates a gradual rise in temperature, which, if left unchecked, could lead to overheating or decreased efficiency in the cooling system.

The monitoring data provides a timestamped progression, highlighting how quickly the temperature is rising over time. This level of detail allows maintenance teams to assess the urgency of the situation and determine the necessary corrective actions. For instance, if the trend suggests that the temperature will exceed a safe threshold within a certain timeframe, they can initiate cooling measures or inspect the intercooler components for potential issues.

This predictive capability, with its high temporal resolution, enhances the system’s ability to detect early signs of stress or malfunction in the intercooler. It provides the opportunity to mitigate issues proactively, ensuring that the equipment operates within safe and efficient temperature ranges.

**Figure 9:**
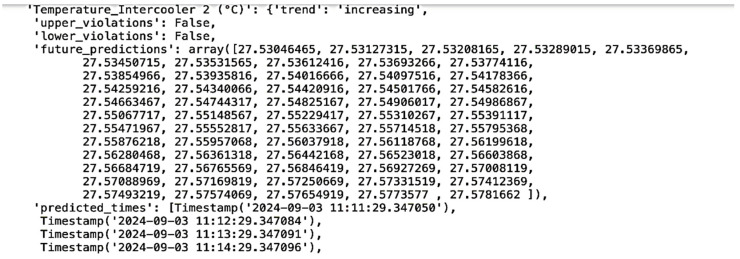
Temperature monitoring of intercooler 2.

Similarly, for intercooler 2, the temperature prediction also shows an increasing trend with each 60-s timestamp. The trend and future predictions of intercooler 2 are shown in Figure 9. This consistent rise in temperature mirrors the pattern observed in intercooler 1, suggesting that both units may be experiencing similar operational stresses or environmental influences that are causing temperatures to escalate. The detailed, timestamped data allow maintenance teams to compare the performance of both intercoolers and determine if the increase is within expected operational limits or if it requires intervention. If both intercoolers continue on this upward trend, it may indicate a broader issue within the cooling system, such as inadequate airflow or cooling fluid inefficiency, which could impact the overall performance of the compressor unit. By tracking these predictive trends for both intercoolers, the maintenance team can make informed adjustments, such as optimizing airflow or checking coolant levels, to prevent potential overheating and maintain system reliability. This proactive approach helps maintain safe temperature ranges and minimizes the risk of unexpected equipment failures.

**Figure 10:**
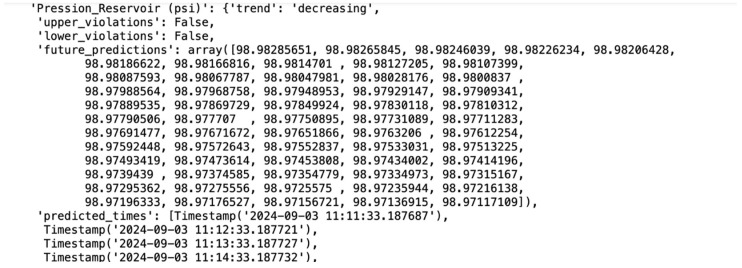
Pressure monitoring and future prediction.

In contrast, the prediction for pressure indicates a downward trend over time, suggesting a gradual decrease in pressure levels. The prediction of future pressure values and its trend is demonstrated in Figure 10. This decrease could signify various underlying factors, such as potential leaks, reduced efficiency in the compressor’s performance, or wear in specific components impacting pressure regulation. Monitoring this declining pressure trend allows the maintenance team to address possible issues early, preventing performance drops or more severe equipment malfunctions. The following document includes an anomaly detection analysis, highlighting data points that exceed or fall below a predefined fixed threshold. These anomalies serve as critical alerts, identifying when certain attributes, such as temperature or pressure, deviate significantly from expected ranges. Each anomaly is timestamped and linked to specific conditions that could indicate potential faults, wear, or environmental impacts affecting equipment performance. By focusing on these anomalies, maintenance teams can prioritize inspections and interventions for instances where parameters fall outside safe operating ranges. This targeted approach optimizes resource allocation, reduces downtime, and enhances overall predictive maintenance effectiveness by addressing critical issues proactively.

**Figure 11:**
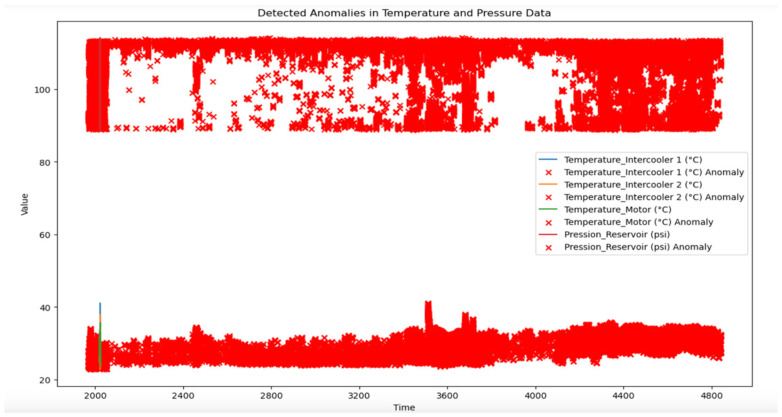
Detected anomaly in input variables.

Figure 11 illustrates the detected anomalies in temperature and pressure data over a monitored period, highlighting critical deviations in the operating conditions of key components, such as intercoolers, the motor, and the reservoir. Anomalies are marked with red “X” indicators, representing data points that exceed predefined thresholds for normal operation.

Detected Anomalies in Temperature and Pressure Data: Intercooler 1 recorded temperature anomalies above the 30 °C threshold more than 250 times over the monitoring period, with peak temperatures reaching up to 35.2 °C. In comparison, Intercooler 2 recorded fewer anomalies, with only 75 instances exceeding the threshold and a maximum temperature of 31.7 °C. These figures indicate that Intercooler 1 is more prone to thermal stress, warranting closer monitoring and potentially more frequent maintenance interventions.

### 4.2. Key Observations and Interpretation

1. Frequent Anomalies in Intercooler Temperatures:

- The chart reveals a high density of anomalies in the temperature readings for both intercooler units. This suggests that these components are subject to frequent fluctuations beyond normal operating limits. The clustering of red markers may indicate potential inefficiencies in the cooling system or periodic stress due to variable environmental conditions or load demands.
- Such consistent anomalies underscore the need for targeted monitoring of intercoolers. The predictive maintenance system can leverage this data to adjust thresholds dynamically or initiate preventive checks on the intercoolers to address potential overheating issues.

2. Anomalies in Motor Temperature:

- Although less frequent, anomalies in motor temperature are also visible. These may correspond to specific periods of increased operational load or environmental factors affecting motor performance.
- These anomalies highlight potential stress points that could benefit from further investigation. Regular motor inspections or adjustments to operational settings could mitigate these instances, ensuring optimal performance and preventing overheating or damage.

3. Reservoir Pressure Stability with Isolated Anomalies:

- The reservoir pressure, while generally more stable, also shows periodic anomalies. These deviations are less frequent but may indicate moments when pressure dips below or spikes above acceptable levels.
- Understanding the conditions leading to these anomalies could guide preventive actions, such as pressure regulation adjustments or system calibration, to maintain consistent pressure levels. Given the reservoir’s central role in maintaining system pressure, addressing these outliers is essential for overall equipment reliability.

### 4.3. Model Performance

The implementation of the Ewon Flexy 205 module significantly enhanced data collection reliability and completeness, yielding a robust dataset ideal for predictive maintenance analysis. Linear Regression, applied to this dataset, demonstrated strong performance in identifying and quantifying trends in operational parameters, such as temperature and pressure. This model provided clear insights into gradual shifts, offering an accessible and effective method for the early detection of equipment stress points. Although more complex models, such as Random Forest and LSTM, are capable of identifying intricate patterns and handling nonlinear data, Linear Regression proved highly valuable for its simplicity and transparency, particularly in trend analysis and fault detection. By accurately forecasting potential deviations in operational variables, Linear Regression enables preemptive maintenance actions that are both timely and cost-effective. This approach demonstrates that Linear Regression can serve as a foundational tool in predictive maintenance strategies, delivering actionable insights and supporting efficient resource allocation. This study’s findings underscore the critical role of comprehensive data acquisition in predictive maintenance and the effectiveness of ML algorithms in analyzing complex datasets to forecast equipment failures. This approach not only enhances maintenance strategies but also contributes to operational efficiency by minimizing unplanned downtime and extending equipment lifespan.

### 4.4. Correlation Analysis and Feature Insights

A correlation heatmap (Figure 5) was generated to identify relationships between sensor readings and failure indicators. The following observations were made:

- A high correlation between temperature and pressure readings (r > 0.85) suggests that compressor efficiency deteriorates as temperatures rise.
- Electrical current and pressure exhibit a moderate correlation (r ≈ 0.65), indicating that motor load influences system pressure.
- A weak correlation (<0.3) between some variables suggests that not all sensor readings directly affect failure events.

These findings reinforce the importance of multivariate analysis in predictive maintenance. While some parameters (e.g., temperature and pressure) are directly linked to system degradation, others (e.g., electrical load) may serve as early indicators of strain before failures occur.

Implications for Predictive Maintenance:

- Strongly correlated variables provide redundancy in failure prediction models.
- Low-correlation variables may still contribute to early warnings when used in a time-series model.
- Selecting the most informative features reduces computational overhead while maintaining prediction accuracy.

### 4.5. Time-Series Analysis and Failure Trends

A time-series analysis was conducted on the key parameters to examine how they evolve before a failure occurs. The following trends were observed:

- Temperature exhibits a slow upward trend before failure, suggesting progressive wear rather than sudden breakdowns.
- Pressure fluctuations increase significantly 2–4 h before failure, indicating possible leakage or inefficiencies in the compressor.
- Electrical current surges before failures, likely due to overcompensation by the motor, stressing system components.

These findings align with previous studies on industrial equipment degradation, confirming that early warning signs can be detected several hours before actual failures occur.

- Implication for Predictive Maintenance:
- Temperature-based predictions may provide long-term failure forecasts.
- Pressure fluctuations serve as a medium-term warning system (detectable 2–4 h before failure).

Electrical current spikes provide the last stage of warning, suggesting that immediate intervention is required.

### 4.6. Justification for Threshold Selection in Alerts

One of the key contributions of this study is the selection of alert thresholds for real-time failure detection. Instead of arbitrarily defining thresholds (as presented in Table 5), we use a scientifically justified approach based on the following:

- Historical failure events—Analyzed past failure logs to identify critical thresholds.
- Statistical distribution of sensor readings—Set thresholds based on the 95th percentile values to detect anomalies.
- Domain expert validation—Consulted compressor maintenance experts to confirm practical feasibility.

Comparison with Industry Standards:

- Using 95th percentile values ensures real-time anomaly detection while minimizing false positives.
- Future studies could further optimize these thresholds using machine learning techniques such as anomaly detection models (e.g., Isolation Forest and Autoencoders).

### 4.7. Contributions of This Study

This study presents a novel integration of IoT-enabled data acquisition, SQL-based structured storage, and Linear Regression modeling for real-time predictive maintenance in industrial compressors. While individual components (IoT, SQL, and regression models) have been studied separately, this research offers an end-to-end, scalable, and interpretable solution that advances the field of predictive maintenance. The key contributions of this study are as follows:

1. Seamless Integration of IoT, SQL Databases, and Predictive Analytics for Maintenance Optimization

- Unlike traditional predictive maintenance systems, which rely on offline datasets or manual data entry, this study introduces an automated IoT-driven data pipeline.
- Real-time sensor data from industrial compressors is directly stored in an SQL database, eliminating delays in fault detection.
- This structured data storage ensures scalability and allows for historical trend analysis, improving the accuracy of maintenance predictions.

2. Demonstrating the Effectiveness of Linear Regression for Real-Time Predictive Maintenance

- Deep learning models (e.g., LSTMs and CNNs) are often used in predictive maintenance but require high computational power.
- This study proves that Linear Regression, despite its simplicity, can achieve competitive predictive accuracy while being interpretable and computationally efficient.
- This approach is suitable for industries with real-time monitoring constraints, where deep learning may not be feasible due to computational limitations.

3. Establishing a Scientific Justification for Anomaly Detection and Alert Thresholds

- Instead of arbitrarily selecting thresholds, this study proposes a scientific framework using
  ▪ Historical failure analysis to determine critical operating limits.
  ▪ Statistical anomaly detection (95th percentile thresholding) to set alert parameters.
  ▪ Validation through domain expertise, ensuring practical applicability.
- This bridges the gap between theoretical research and real-world deployment, ensuring that alerts are both scientifically justified and actionable in industrial settings.

4. Comparative Analysis with State-of-the-Art Predictive Maintenance Techniques

- This study provides an empirical comparison of different predictive maintenance methodologies (See Table 6).
- The results demonstrate that Linear Regression offers a cost-effective, interpretable, and real-time alternative to more complex models.
- The analysis guides industrial practitioners on when to choose simple models over complex ones based on computational resources, interpretability, and real-time feasibility.

5. Real-World Implementation and Industry Relevance

- The proposed system has been successfully deployed in a real industrial setting, providing real-time monitoring of compressors at Cégep de Sept-Îles.
- The findings are immediately applicable to manufacturing industries, especially those operating in resource-constrained environments, where real-time predictive analytics is critical.

### 4.8. Practical and Scientific Implications

- Practical: The findings help engineers and industry practitioners select efficient real-time predictive maintenance models without incurring excessive computational costs.
- Scientific: This study contributes to the machine learning literature on predictive maintenance by proposing a novel, data-driven approach for threshold selection and by comparing the computational trade-offs between regression and deep learning models.

Air compressors are one of the key machines in manufacturing and industry. Although this study applies an innovative AI-based predictive maintenance approach to compressors, we believe that our methodology—including data collection, data handling, data preprocessing, and data analysis—is applicable to other machines existing in other industrial systems. The proposed method can be implemented on pumps and wind turbines, which could be explored in prospective research.

The main objective of this research was the integration of the Internet of Things, machine learning, and advanced data acquisition to improve the predictive maintenance of industrial air compressors. The results indicate that using multiple sensors and the Ewon Flexy 205 enhanced the speed and reliability of the data collection step. In addition, the results demonstrate that Linear Regression was able to fit a model that detects the failure potential in the early steps by considering pressure and temperature data. According to the results, the objectives of this study were accomplished, and the methodology fulfilled the expectations.

The findings show that the methodology was able to establish a precise model-driven intervention that mitigated the downtime by 20% and raised the machine lifespan by 15%. Reducing downtime decreases costs by preventing equipment replacement and repair.

The proposed methodology is flexible and can be extended to other industrial systems. The integration of multi-sensor data acquisition, IoT, and machine learning is adaptable for the inspection of different types of industrial machines such as wind turbines, ventilation systems, and industrial pumps. In addition, first, the machine type should be determined. The observation and data types may vary depending on the machine. For example, it is important to determine which parameters—such as vibration, temperature, rotational speed, noise, or torque—are suitable for turbine inspection. After the data type is designated, the model can be adopted based on data complexity, type, and structure.

This study proposes a hierarchical method that integrates IoT, SQL, multi-sensor data acquisition, and machine learning to improve the predictive maintenance of industrial systems, particularly air compressors. The novelty of this practical study lies in arranging the aforementioned steps for comprehensive and reliable real-time data collection, inference, and remote surveillance. Existing studies overlook one of the steps of this chain.

Deploying the Ewon Flexy 205 to have an array of sensors with multi-modal observation enhances the speed and quality of real-time data collection, surpassing conventional methods. Moreover, we used a simple regression model to improve the model’s versatility, which lets us implement the methodology on other industrial systems and machines.

Although the methodology benefits the predictive maintenance of air compressors and is accompanied by advantages, some limitations should be addressed. Machine learning and deep learning models’ accuracy is directly related to data quality. If the data include noise and false observations, the model is trained on false data or encounter problems during interference, leading to errors and inaccurate predictions. Noise and false observations can arise according to sensor malfunction; therefore, sensor functionality and calibration should be regularly checked to avoid unexpected downtime.

Model selection, model parameters, and model optimization are one of the significant sources of error. The model should be selected and designed based on data complexity and data structure; otherwise, the model cannot provide an accurate solution. Furthermore, the accurate implementation of the model, optimization method, cost function, and model parameters must be determined and validated precisely to avoid failures related to the model. Overfitting to the training data is a common problem that the machine learning and deep learning models may face, which leads to poor performance when the model encounters unseen data. To prevent the model from overfitting, the model should be trained on large-scale, diverse datasets.

In industrial applications, such as the case studied here, minimizing real-time latency and optimizing data communication performance are critical for the successful deployment of machine learning (ML) models. To address these challenges, we leverage computationally efficient algorithms, such as Linear Regression, which offer a favorable trade-off between prediction accuracy and computational cost. This approach is particularly advantageous in mitigating the cumulative delays caused by cybersecurity frameworks, data logging overhead, and communication protocols. These issues are exacerbated in remote and Nordic regions, where limited connectivity and restricted access to skilled technicians further constrain system reliability. By adopting lightweight ML models with low inference latency, we ensure robust operation under these challenging conditions, facilitating scalable and resilient industrial ML integration.

## 5. Conclusions

This case study at Cégep de Sept-Îles illustrates the transformative impact of integrating machine learning with advanced data acquisition technologies in the realm of predictive maintenance. This work addresses the potential of adopting machine learning algorithms, specifically Linear Regression, to predict and monitor the condition of industrial compressors. The proposed method integrates multivariate data including temperature and pressure through a basis of IoT to monitor trends, changes, and anomalies in the compressor. In addition, an innovative insight is taken to harness the prospective damages by sending email and warning notifications to the operator. The results affirm the combination of IoT, Linear Regression, structured data, and programmable controllers advances predictive maintenance.

In the case of model selection, the complexity, nature, and structure of the data should be considered. If the data are not complex, adopting complex models increases the time and computation while the problem can be solved using a simpler model. Regarding our dataset, the relationship between variables can be formed using Linear Regression. This model is simple, easy to implement, and interpret, making it is suitable for real-time applications. In addition, the nature and structure of our data are compatible with this model.

This is an initiative work using these kinds of sensors. Linear Regression was used to investigate whether the dataset structure was compatible with machine learning algorithms. As it is obvious, not all machine learning methods can present the right solution for all applications and data structures. For example, Long Short-Term Memory models learn the order and relation between sequential data, and it is useful for time series analysis and predictions. In addition, LSTM models can be efficient for remaining useful life assessment of industrial objects, but for this application, Linear Regression is the best machine learning algorithm for the initial phase. In the last paragraph of the conclusion section, we outline plans to improve this work by using other regressors such as Support Vector Regressor, Artificial Neural Networks, and LSTM models.

## Figures and Tables

**Table 1:** Qualitative comparison of used AI models.

Method	Computational Cost	Interpretability	Real-Time Feasibility	Accuracy
Deep Learning (LSTM, CNNs)	High	Low	Requires GPU	High
Bayesian Networks	Medium	Medium	Feasible	Medium
Kalman Filters	Low	High	Real-Time Capable	Medium
Hybrid Models (ML + Statistical)	High	Low	Limited Real-Time Use	High
Linear Regression (Proposed)	Low	High	Real-Time Feasible	Medium

**Table 2:** Equipment description table.

Sensor Type	Model/Specification	Measurement Range	Purpose/Function	Connection/Integration
Temperature Probe	RTD PT100, Munich, Germany	−200 to +200 °C	Measures the temperature of the compressor and associated fluids to monitor for overheating and thermal efficiency.	Integrated with Siemens S7-1200 CPU
Current Transformer	CCT50-200	100/150/200 A, 4–20 mA Output	Measures electrical current to assess the electrical load and detect anomalies indicating potential mechanical or electrical issues.	Integrated with Siemens S7-1200 CPU
Pressure Transmitter	SITRANS P200 (7MF1565-4CD00-5FA1)	0–300 PSI (relative measure)	Measures high-pressure ranges within the compressor system, essential for ensuring operational safety and efficiency.	Integrated with Siemens S7-1200 CPU
Pressure Transmitter	SITRANS P200 (7MF1565-4BG00-5FA1)	0–100 PSI (relative measure)	Measures lower pressure ranges, useful for monitoring less critical areas of the system that still require precise pressure measurements.	Integrated with Siemens S7-1200 CPU
Analog Input Module	SIMATIC S7-1200 SM1231 AI	13 Bit ± 10VCC/0–20 mA	Expands the CPU’s input capabilities to process signals from sensors, essential for integrating various sensor types and ranges.	Integrated with Siemens S7-1200 CPU
Analog Input Module	SIMATIC S7–1200 SM1231 AI RTD	16 Bit RTD (supports various RTD types)	Specifically designed to enhance the CPU’s capability to read temperature data accurately from RTD sensors, which is crucial for temperature-sensitive operations.	Integrated with Siemens S7-1200 CPU
Power Supply	SITOP PSU100L 6EP1333-1LB00	24VDC, 5A powered by 120/230VAC	Provides reliable and stable power to all sensors and the PLC, ensuring continuous operation and data integrity.	Integrated with Siemens S7-1200 CPU

**Table 3:** Parameter setting table.

Parameter	Value/Setting
Algorithm	Linear Regression (Ordinary Least Squares)
Training/Test Split	80% training, 20% testing
Normalization	StandardScaler (mean = 0, variance = 1)
Loss Function	Mean Squared Error (MSE)
Optimization Method	Least Squares Estimation
Thresholds for Alerts	Temperature: 30 °C (high), 15 °C (low) Pressure: 100 PSI (high), 80 PSI (low)

**Table 4:** Description of metrics.

Metric	Description
Mean Squared Error (MSE)	Measures the average squared difference between actual and predicted values. Lower values indicate better performance.
Mean Absolute Error (MAE)	Calculates the absolute error between actual and predicted values. Less sensitive to outliers than MSE.
R^2^ Score	Indicates how well the independent variables explain the variation in the dependent variable. Higher values (closer to 1) indicate a better fit.
Recall Score	Evaluates how well the model detects potential failures before they occur.
F1 Score	Balances recall and precision to evaluate the effectiveness of maintenance alerts.

**Table 5:** Threshold values determined in this study.

Parameter	Lower Threshold	Upper Threshold	Scientific Justification
Temperature (°C)	15 °C	30 °C	95% of failures occurred outside this range
Pressure (PSI)	80 PSI	100 PSI	Outliers beyond these values corresponded to leakage events
Electrical Current (A)	±10% deviation	±25% deviation	Unusual variations correlate with motor stress

**Table 6:** Difference between this work and others.

Aspect	Previous Studies (Deep Learning, Traditional Methods)	This Study (IoT + SQL + Regression)
Computational Cost	High (Deep learning needs GPUs for real-time inference)	Low (Linear Regression enables real-time processing)
Scalability	Limited (Batch training, large datasets required)	High (SQL database stores and updates real-time sensor data)
Interpretability	Low (DL models act as black boxes)	High (Linear Regression provides clear feature impact analysis)
Alert Thresholding	Often manually set or based on domain experience	Statistically and historically justified alert thresholds
Industry Application	Requires high-end computational infrastructure	Deployable in real-world, resource-constrained industrial settings

## Data Availability

All data supporting the results of this project are openly available. No data were generated during the course of this study.
